# Genetic polymorphism study at 15 autosomal locus in central Indian population

**DOI:** 10.1186/s40064-015-1364-1

**Published:** 2015-09-30

**Authors:** Pankaj Shrivastava, Toshi Jain, Veena Ben Trivedi

**Affiliations:** DNA Fingerprinting Unit, State Forensic Science Laboratory, Department of Home (Police), Govt. of MP, Sagar, 470001 India

**Keywords:** DNA typing, Autosomal STR, Central India, Population data, Forensic

## Abstract

The analysis of 15 autosomal STR locus (TH01, D3S1358, vWA, D21S11, TPOX, D7S820, D19S433, D5S818, D2S1338, D16S539, CSF1PO, D13S317, FGA, D18S51, D8S1179) was done in 582 healthy unrelated individuals (Male-366, Female-216) originating from the various geographical regions of Madhya Pradesh, India. All locus fall under Hardy–Weinberg equilibrium except TPOX. These STR loci were highly informative and discriminating with combined power of discrimination (CPD) >0.99999. Locus wise allele frequencies of the studied population were compared with the other published populations. Also the Clustering pattern and genetic distance of studied populations is compared and presented with various populations. The studied population showed the genetic proximity with geographically close populations of India and significant genetic variation with distant populations which is also evident by clustering pattern of the NJ tree and the PCA plot.

## Background

After almost 30 years since the first formal application of DNA technology (Jeffreys et al. 1985), short tandem repeats (STR’s) based DNA analysis (Edwards et al. 1992) was accepted as a core method in forensics, it is still being routinely used in cases of simple paternity testing (Zupanic Pajnic et al. 2001), identification of human remains testing (Zupanic Pajnic et al. 2010) and in complicated criminal casework analysis, including rape and mass rape. STR’s form approximately 3 % of the total human genome and on an average are present once in every 10,000 nucleotides (Butler 2005). Due to ease of use due to multiplexing, these markers are routinely used in forensic, anthropological and medical studies. With the growing number of laboratories using STR analysis technology, more and more population STR data have been reported (Tandon et al. 2002; Sarkar and Kashyap 2002; Sahoo and Kashyap 2002; Gaikwad and Kashyap 2002; Rajkumar and Kashyap 2004; Narkuti et al. 2008; Dubey et al. 2009; Ghosh et al. 2011; Chaudhari and Dahiya 2014; Giroti and Talwar 2010; Shrivastava et al. 2015a; b).

India is the largest secular country with a polygenetic population. Various known religions are found in India and the Indian population belongs to various linguistic and ethnic groups of different castes and tribes and it is said to be the melting pot of various ethnic groups (Eaaswarkhanth et al. 2009). Human diversity in India is defined by 4693 differently documented population groups that include 2205 major communities, 589 segments and 1900 territorial units spread across the country (Singh 1998). Major population migrations, social structure and caste endogamy has influenced the genetic structure of Indian populations.

Madhya Pradesh, a state in Central India is the second largest state in the country by area. Population of Madhya Pradesh is 72,597,565 comprising 37,612,920 males and 34,984,645 females, contributing 6 percent to India’s total population (Census of India 2011). With these rationales 15 highly polymorphic autosomal microsatellite markers including 13 core forensic loci, have been analyzed and the distribution of alleles across various populations is compared with the previously published data on the same markers from different parts of India (caste specific available data) and other area specific reported data only one from India and rest from other parts of world, in order to decipher genetic delineation amongst the populations (Tables 1, 2). As the genetic data being reported here is area specific therefore, besides comparing the data with the population geographically close (caste specific) to the population of Madhya Pradesh and other parts of India, the data was also compared with the area specific available data.

**Table 1:** Population data used for analysis using NJ tree and PCA plot to observe genetic distance with other reported Indian populations

S. no.	Published population	Number of loci	References
1	Balmiki (Punjab)	15	Ghosh et al. (2011)
2	Sakaldwipi Brahmin (Jharkhand)	15	Ghosh et al. (2011)
3	Konkayastha_Brahmin (Maharashtra)	15	Ghosh et al. (2011)
4	Mahadev Koli (Maharashtra)	15	Ghosh et al. (2011)
5	Iyengar (Tamilnadu)	15	Ghosh et al. (2011)
6	Kurumans (Tamilnadu)	15	Ghosh et al. (2011)
7	Tripuri (Tripura)	15	Ghosh et al. (2011)
8	Riang (Tripura)	15	Ghosh et al. (2011)
9	Munda (Jharkhand)	15	Ghosh et al. (2011)
10	Adi_Minyong (Arunachal Pradesh)	15	Krithika et al. (2007)
11	Chenchu (Andhra Pradesh)	15	Bindu et al. (2005)
12	Lambadi (Andhra Pradesh)	15	Bindu et al. (2005)
13	Naikpod_Gond (Andhra Pradesh)	15	Bindu et al. (2005)
14	Yerukula (Andhra Pradesh))	15	Bindu et al. (2005)
15	Munda (Chotanagpur)	15	Banerjee et al. (2005)
16	Santal (Chotanagpur)	15	Banerjee et al. (2005)
17	Oraon (Chotanagpur)	15	Banerjee et al. (2005)
18	Lodha (Bengal)	15	Singh et al. (2006)
19	Kora (Bengal)	15	Singh et al. (2006)
20	Maheli (Bengal)	15	Singh et al. (2006)
21	Adi_Pasi (Arunachal Pradesh)	15	Krithika et al. (2007)
22	Bhil (Madhya Pradesh)	15	Shrivastava et al. (2015b)
23	Bhil (Gujrat)	15	Chaudhari and Dahiya (2014)

**Table 2:** Population data used for analysis using NJ tree and PCA plot to observe genetic distance with other reported area specific populations

S. no.	Published populations	Number of loci	References
1	Palestinian_Iraq population	15	AL-Zubaidi et al. (2014)
2	Swedish population	15	Montelius et al. (2008)
3	Hungarian population	15	Demeter et al. (2010)
4	Moroccan population	15	Bentayebi et al. (2014)
5	Shenyang_China population	13	Hou et al. (2013)
6	Wallachia_Romania population	15	Stanciu et al. (2009)
7	Guangdong population	15	Zhu et al. (2005)
8	Tamil population	15	Balamurugan et al. (2010)
9	Croatian population	15	Projić et al. (2007)
10	Estonian population	15	Sadam et al. (2014)
11	Latvia population	10	Jemeljanova et al. (2015)

## Methods

### The population and DNA extraction

The population sample consisted of 582 healthy, unrelated individuals (Male-366, Female-216) originating from different geographical regions of Madhya Pradesh. Samples were taken from routine casework performed by the first author at the DNA fingerprinting Unit, State Forensic Science Laboratory, Sagar, Madhya Pradesh, India from the period of 2007 to 2013 after written informed consent. Only fathers and mothers were selected from paternity trios and unrelated individuals were taken into consideration from complex kinship analyses. DNA was extracted from the peripheral blood samples by automated DNA extraction system 12 GC (Precision System Science Co., Ltd., Matsudo, Japan).

### DNA quantitation

Real Time PCR ABI 7000 (Applied Biosystems, Foster City, CA, USA) was used for quantification of the isolated DNA using the Quantifiler DNA Quantification Kit (Applied Biosystems, Foster City, CA, USA) as per the recommended protocol by the manufacturer.

### Amplification

1 ng of DNA template was used to simultaneously amplify 15 STR locus including 13 CODIS (D3S1358, TH01, D21S11, D18S51, D5S818, D13S317, D7S820, D16S539, CSF1PO, vWA, D8S1179, TPOX, FGA) and 2 additional loci (D2S1338 and D19S433), as well as the gender determining locus Amelogenin using AmpFlSTR Identifiler or AmpFlSTR Identifiler Plus kit (Applied Biosystems, Foster City, CA, USA). Similar amount of DNA was used in all PCR reactions. Amplification was carried out according to the manufacturer’s recommended protocol, with a modification of decreasing the total volume of each reaction to 12.5 μL. The PCR amplification was carried out in AB Gene Amp PCR System 9700 Thermal Cycler (Applied Biosystems, Foster City, CA, USA).

### Typing

Multicapillary electrophoresis of the amplification products was performed on an ABI Prism 3100 Avant Genetic Analyzer (Applied Biosystems, Foster City, CA, USA) using LIZ 500 size standard (Applied Biosystems, Foster City, CA, USA) provided with the kit and the data was analysed using GeneMapper™ 3.5 Software (Applied Biosystems, Foster City, CA, USA). All steps were done according to the Laboratory’s internal control standards and respective kit controls, according to the IFSH recommendations (DNA recommendations 1994).

### Quality control

Passed Proficiency testing of the GITAD, Spain http://gitad.ugr.es/principal.htm). Also, laboratory internal control standards and kit controls used.

### Analysis of data

Allele frequency of the 15 STR loci was calculated by GenAlEx 6.5 software (Peakall and Smouse 2006). Several forensic parameters, i.e., polymorphism information content (PIC), power of discrimination (PD), power of exclusion (PE), matching probability (P_m_) and paternity index (PI) was calculated using the PowerStatsV1.2 spreadsheet program (Tereba 1999). Observed heterozygosity (H_obs_), Expected Heterozygosity (H_exp_) and Hardy–Weinberg equilibrium (HWE) using exact test was calculated using Arlequin v3.5 (Excoffier et al. 2005). Allele frequencies of studied population were compared with other published populations using Fst pair wise distance by Arlequin v3.5 software (Excoffier et al. 2005). Nei’s genetic distances (Nei 1972) among compared populations were derived and subsequently used to generate a Neighbour joining (NJ) dendrogram using POPTREE2 program (Takezaki et al. 2009). The robustness of the phylogenetic relationship established by the NJ tree was assessed using bootstrap analysis with 1000 replications. Graphical representation of genetic distances was also performed based on Principle component analysis (PCA) plot using PAST 3.02a software (Hammer et al. 2001).

## Results and discussion

The genetic variations in the allele-frequency distribution at 15 STR loci and statistical analysis of forensic parameters for the studied populations are shown in Table 3. In total, 158 alleles were observed in the central Indian population with corresponding allele frequencies ranging from 0.001 to 0.381 (Table 3). In which CSF1PO locus was found to have a maximum allele frequency with allele 12 (0.381) being the most frequent allele in this population. Locus wise distribution of the most common and least common allele in studied population is summarized and presented in Table 4. The peak high threshold was 50 RFU for heterozygous and 200 RFU for homozygous alleles. The combined power of exclusion (CPE) and combined power of discrimination (CPD) for all 15 STR locus were 0.9999 and greater than 0.99999 respectively in studied population. The combined matching probability was found to be 1.51 × 10^18^. Among all the studied locus, no significant deviations from Hardy–Weinberg expectations were observed even after Bonferroni correction (Bland and Altman 1995) except at locus TPOX (p < 0.003). At TPOX locus all the homozygotic peaks were found with a peak height of more than 200 RFU, thus removing the possibility of any heterozygous peak. Allele 11(437 out of 1164) followed by allele 8 (410 out of 1164) were found to be the dominating alleles in this population.

**Table 3:** Observed allele frequency and forensic parameters for 15 autosomal loci in central Indian population (N = 582)

Allele/n	D8S1179	D21S11	D7S820	CSF1PO	D3S1358	THO1	D13S317	D16S539	D2S1338	D19S433	VWA	TPOX	D18S51	D5S818	FGA
6						0.282									
7			0.021	0.003		0.154						0.003			
8	0.005		0.234	0.005		0.127	0.220	0.085				0.352		0.001	
9	0.007		0.067	0.030		0.293	0.107	0.149		0.001		0.145	0.001	0.023	
9.3						0.138									
10	0.168		0.232	0.193		0.006	0.090	0.093		0.003		0.095	0.003	0.124	
11	0.072		0.247	0.294			0.253	0.315		0.004		0.375	0.027	0.337	
11.2				0.002											
12	0.109		0.170	0.381	0.001		0.246	0.227		0.074		0.027	0.068	0.314	
12.2										0.004				0.005	
13	0.166		0.028	0.079	0.002		0.070	0.114		0.290		0.003	0.129	0.186	
13.2										0.017					
14	0.199		0.001	0.013	0.045		0.015	0.014		0.245	0.108		0.280	0.011	
14.2										0.060					
15	0.176			0.001	0.306			0.003		0.136	0.095		0.169		
15.2										0.087	0.002				
16	0.084				0.302				0.008	0.048	0.224		0.144		
16.2										0.021	0.001				
17	0.013				0.241				0.067	0.009	0.258		0.076		
17.2										0.002	0.001				
18	0.001				0.097				0.158		0.204		0.040		0.007
18.2											0.001				
19					0.008				0.144		0.092		0.033		0.049
20									0.131		0.013		0.015		0.107
20.2															0.005
21									0.053		0.001		0.008		0.143
21.2															0.004
22									0.069				0.004		0.152
22.2															0.012
23									0.168				0.001		0.183
23.2															0.009
24									0.099				0.001		0.156
24.2															0.006
25									0.090						0.121
25.2															0.001
26									0.010						0.030
27		0.008							0.003						0.015
28		0.143													
29		0.209													
29.2		0.004													
30		0.187													
30.2		0.027													
31		0.045													
31.2		0.125													
32		0.008													
32.2		0.182													
33.2		0.048													
34.2		0.012													
35		0.001													
35.2		0.002													
P_m_	0.043	0.041	0.069	0.121	0.108	0.084	0.068	0.070	0.028	0.056	0.063	0.139	0.046	0.112	0.034
PD	0.957	0.959	0.931	0.879	0.892	0.916	0.932	0.930	0.972	0.944	0.937	0.861	0.954	0.888	0.966
PIC	0.831	0.828	0.765	0.678	0.702	0.741	0.774	0.770	0.866	0.796	0.786	0.653	0.824	0.693	0.854
PE	0.699	0.649	0.517	0.419	0.505	0.482	0.566	0.559	0.709	0.591	0.623	0.391	0.672	0.479	0.709
PI	3.384	2.881	2.035	1.635	1.980	1.877	2.291	2.256	3.506	2.445	2.670	1.540	3.096	1.865	3.506
H_obs_	0.852	0.826	0.754	0.694	0.747	0.734	0.782	0.778	0.857	0.796	0.813	0.675	0.838	0.732	0.857
H_exp_	0.850	0.848	0.797	0.725	0.747	0.776	0.804	0.798	0.879	0.819	0.813	0.705	0.842	0.738	0.869
*P*value	0.016	0.488	0.559	0.043	0.292	0.011	0.749	0.038	0.261	0.074	0.079	0.001	0.038	0.327	0.014

*Pm* matching probability, *PD* power of discrimination, *PIC* polymorphism information content, *PE* power of exclusion, *PI* paternity index, *H*_*obs*_ observed heterozygosity, *H*_*exp*_ expected heterozygosity, *P value* HWE test

**Table 4:** The most common allele (MCA) and least common allele (LCA) in studied Central Indian population

Allele	MCA	LCA
D8S1179	14	18
D21S11	29	35
D7S820	11	14
CSF1PO	12	15
D3S1358	15	12
THO1	9	10
D13S317	11	14
D16S539	11	15
D2S1338	23	27
D19S433	13	9
vWA	17	16.2,17.2,18.2,21
TPOX	11,8	7,13
D18S51	14	9,23,24
D5S818	11	8
FGA	23	25.2

*MCA* most common allele, *LCA* least common allele

**Fig. 1:**
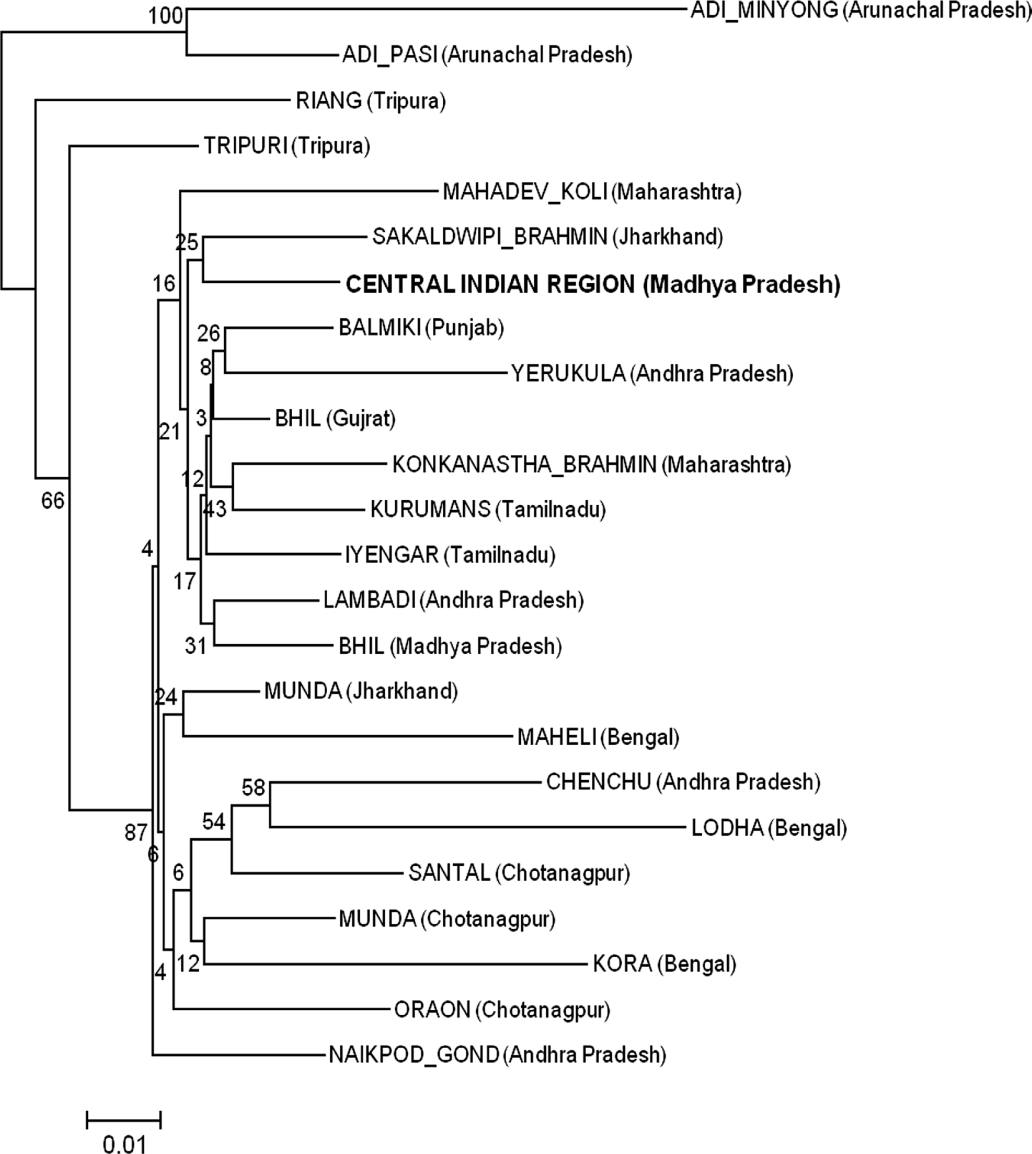
NJ tree based on Nei’s genetic distance showing genetic distance of central Indian population with other reported Indian populations

**Fig. 2:**
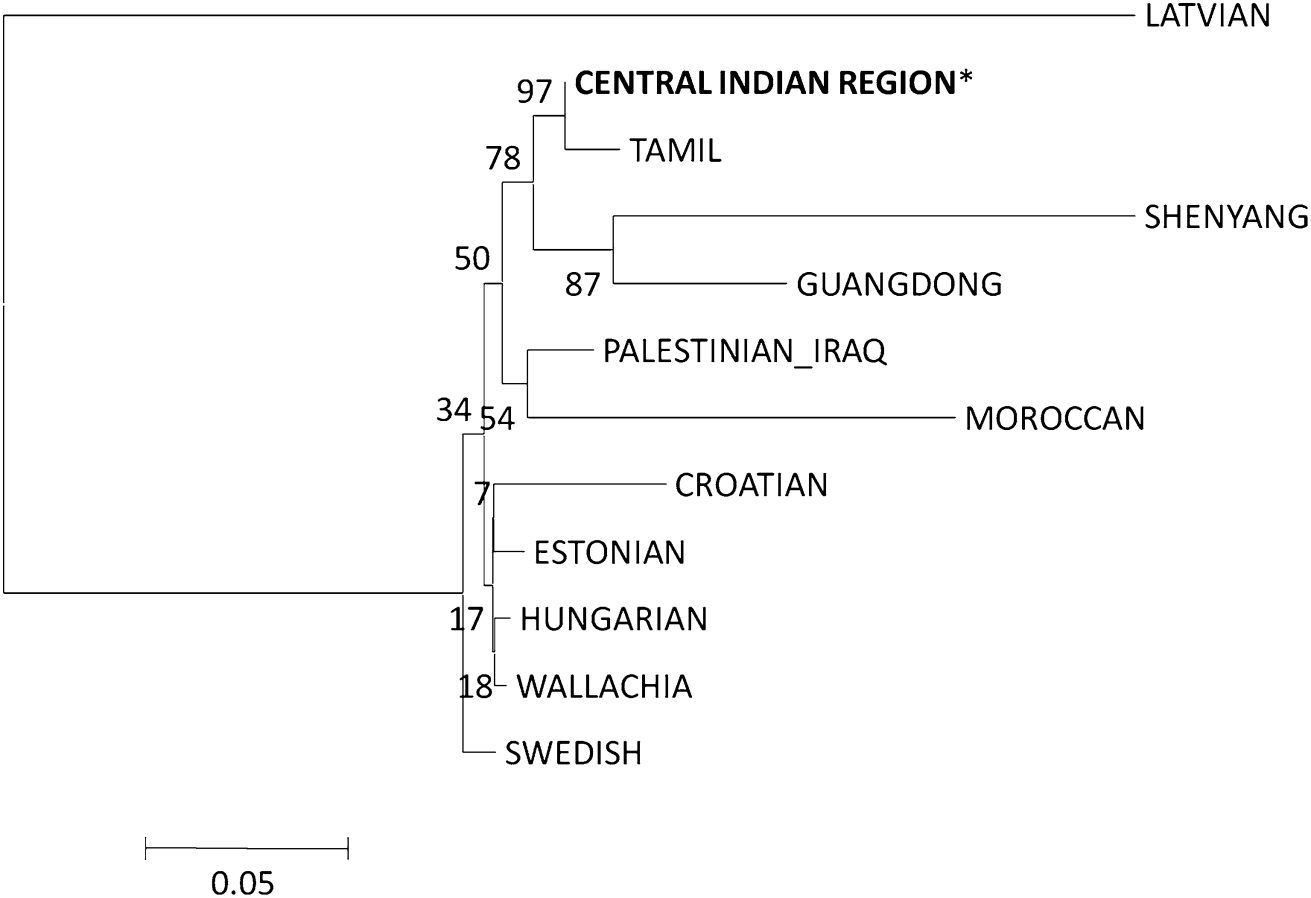
NJ tree for the studied population with other reported populations from different countries based on Nei’s genetic distances

**Fig. 3:**
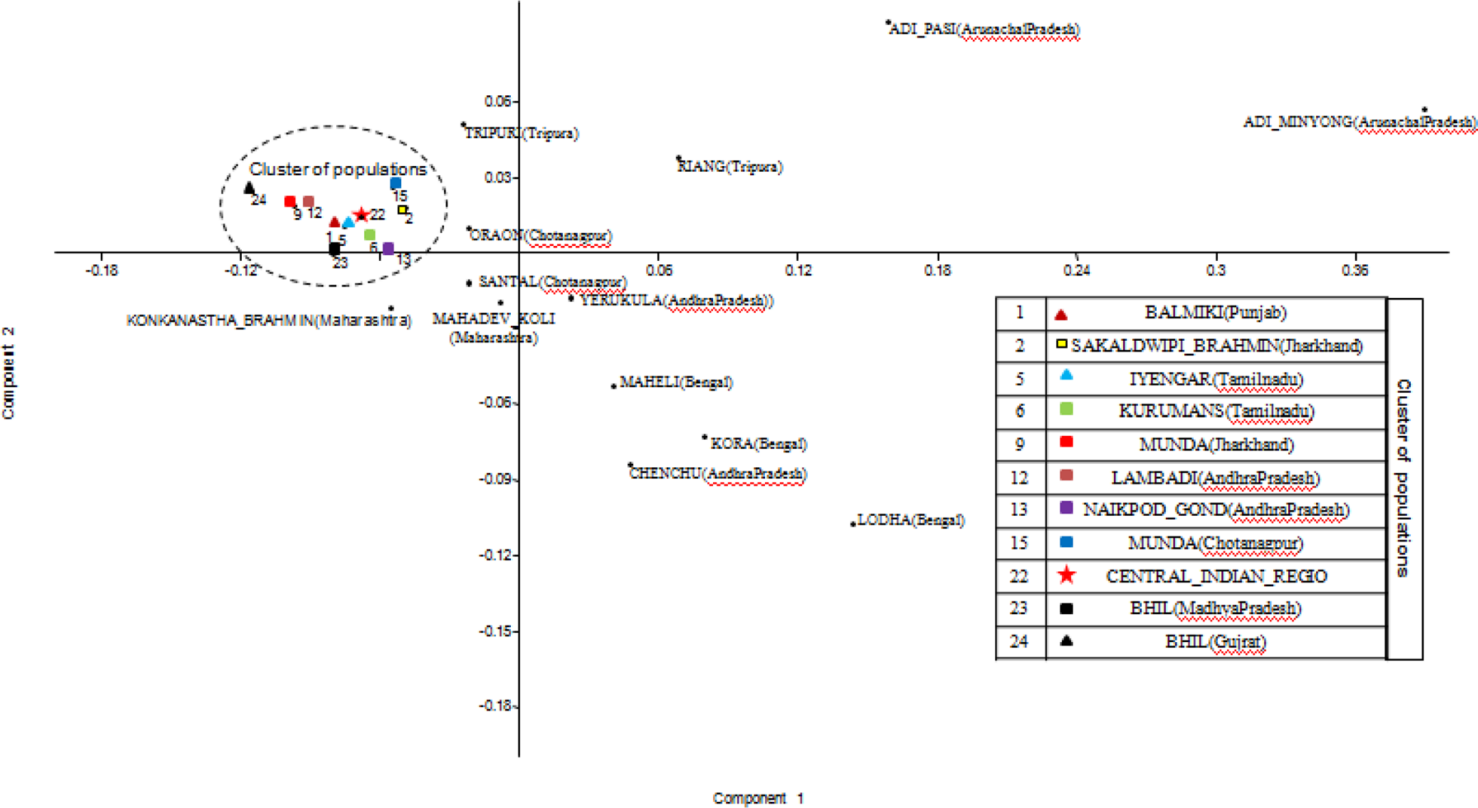
PCA plot of central Indian population showing distance pattern with other published Indian populations of India

The expected heterozygosity and the power of discrimination calculated from allele frequencies obtained from central Indian population revealed that in combination, the 15 autosomal STRs have a high forensic efficacy. Locus wise allele frequencies of studied population were compared at all 15 loci with the other published Indian populations including geographically close populations viz. Chenchu (Andhra Pradesh), Lambadi (Andhra Pradesh), Yerucula (Andhra Pradesh) and Naikpood (Andhra Pradesh) (Bindu et al. 2005), Adimiong (Arunachal Pradesh) and Adipasi (Arunachal Pradesh) (Krithika et al. 2007), Munda (Chotanagpur), Santal (Chotanagpur) and Oraon (Chotanagpur) (Banerjee et al. 2005), Kora (Bengal), Lodha (Bengal) and Maheli (Bengal) (Singh et al. 2006), Bhil (Gujrat) (Chaudhari and Dahiya 2014), Balmiki (Punjab), Sakaldwipi Brahmin (Jharkhand), Munda (Jharkhand), Konkanastha Brahmin (Maharashtra), Mahadev Koli (Maharashtra), Iyengar (Tamilnadu), Kurumans (Tamilnadu), Tripuri (Tripura) and Riang (Tripura) (Ghosh et al. 2011), Bhil (Madhya Pradesh) (Shrivastava et al. 2015b) populations using Pairwise Fst distance ranging from −0.003 to 0.247 (Tables 5, 6, 7, 8). Central Indian population showed a considerable genetic distance with other published indian population which were used for comparison (Table 1). Central Indian population showed significant variation at 14 loci with Lodha (Bengal), at 11 loci with Adi pasi population (Arunachal Pradesh) and Kora population (Bengal), at 10 loci with Yerukula population (Andhra Pradesh) and Maheli population (Bengal), at 9 loci with Adiminyong population (Arunachal Pradesh), at 8 loci with Naikpod Gond population (Andhra Pradesh) and Oraon population (Chotanagpur), at 7 loci with Riang population (Tripura) and Munda population (Chotanagpur), at 6 loci with Chencheu population (Andhra Pradesh), Santal population (Chotanagpur) and Mahadev koli population (Maharashtra), at 5 loci with Bhil (Gujrat), at 4 loci with Tripuri population (Tripura), at 3 locus with Munda population (Jharkhand) and Sakaldwipi brahmin population (Jharkhand), at 2 loci with Konkanastha Brahmin population (Maharashtra), Kurumans population (Tamilnadu), Balmiki population (Punjab) and Bhil population (Madhya Pradesh) and at 1 locus with Iyengar (Tamilnadu). Central Indian population showed no significant variation from Lambadi population (Andhra Pradesh). Neighbour Joining dendrogram (Figs. 1, 2) based on Nei’s genetic distance (Nei 1972) showed genetic relationships of the studied population with other Indian published populations. The grouping of populations in PCA plot (Fig. 3) is also found consistent with the clustering pattern observed in the NJ tree.

**Table 5:** Fst pairwise genetic distances and corresponding P value

Central India V/S	Bhil (Madhya Pradesh)		Balmiki (Punjab)		Mahadev Koli (Maharashtra)		Iyenger (Tamilnadu)		Kurumans (Tamilnadu)		Tripuri (Tripura)	
Locus	Fst	P-value	Fst	P-value	Fst	P-value	Fst	P-value	Fst	P-value	Fst	P-value
D8S1179	0.009	0.001	0.013	0.001	0.006	0.025	0.000	0.395	0.000	0.365	0.000	0.363
D21S11	0.001	0.230	−0.001	0.621	0.000	0.479	0.042	0.000	0.001	0.216	0.009	0.008
D7S820	0.000	0.458	−0.002	0.695	0.003	0.150	0.000	0.412	0.006	0.055	0.004	0.104
CSF1PO	−0.002	0.826	0.000	0.374	−0.002	0.624	−0.003	0.742	0.008	0.038	0.001	0.260
D19S433	0.002	0.078	0.000	0.478	0.023	0.000	0.007	0.031	0.001	0.310	0.007	0.015
vWA	0.001	0.268	0.006	0.049	0.018	0.001	0.005	0.047	0.024	0.000	0.018	0.001
TPOX	0.017	0.000	0.000	0.411	0.019	0.003	0.000	0.379	0.017	0.005	0.049	0.000
D18S51	0.005	0.008	0.000	0.452	0.014	0.000	−0.001	0.628	0.012	0.004	0.011	0.005
D3S1358	0.000	0.425	0.011	0.016	0.007	0.052	0.005	0.097	0.004	0.099	0.011	0.005
THO1	0.006	0.011	0.008	0.035	0.005	0.050	0.004	0.101	0.004	0.124	0.066	0.000
D13S317	0.000	0.414	0.006	0.047	0.036	0.000	0.008	0.023	0.000	0.410	−0.002	0.693
D16S539	0.001	0.286	0.025	0.000	0.016	0.000	0.000	0.437	0.012	0.002	0.013	0.002
D2S1338	0.001	0.163	0.000	0.393	0.015	0.000	−0.001	0.746	0.002	0.198	0.008	0.005
D5S818	0.002	0.108	0.006	0.088	0.006	0.045	−0.001	0.502	0.003	0.128	0.002	0.199
FGA	0.002	0.126	0.002	0.183	0.003	0.118	−0.001	0.566	0.006	0.018	0.000	0.350

P value <0.003 values

**Table 6:** Fst pairwise genetic distances and corresponding P value

Central India V/S	Riang (Tripura)		Munda (Jharkhand)		Lambadi (Andhra Pradesh)		Naikpod Gond (Andhra Pradesh)		Yerukula (Andhra Pradesh)		Munda (Chotanagpur)	
Locus	Fst	P-value	Fst	P-value	Fst	P-value	Fst	P-value	Fst	P-value	Fst	P-value
D8S1179	0.006	0.030	0.027	0.000	0.004	0.020	0.030	0.000	0.016	0.000	0.013	0.000
D21S11	0.012	0.001	0.009	0.006	0.001	0.157	0.003	0.048	0.015	0.000	0.015	0.000
D7S820	0.007	0.041	0.009	0.009	0.004	0.035	0.000	0.404	0.014	0.000	0.003	0.090
CSF1PO	0.000	0.354	−0.001	0.507	0.001	0.256	0.003	0.104	0.002	0.179	0.003	0.076
D19S433	0.019	0.000	0.005	0.049	0.000	0.292	0.004	0.044	0.014	0.000	0.000	0.414
vWA	0.029	0.000	0.008	0.013	0.001	0.244	0.008	0.002	0.041	0.000	0.012	0.000
TPOX	0.247	0.000	0.005	0.097	0.002	0.184	0.027	0.000	0.000	0.328	0.008	0.007
D18S51	0.010	0.006	0.004	0.072	−0.001	0.630	0.007	0.004	0.006	0.004	0.002	0.074
D3S1358	0.007	0.042	0.005	0.068	0.010	0.007	0.001	0.270	0.031	0.000	0.003	0.073
THO1	0.065	0.000	0.021	0.000	0.000	0.462	0.042	0.000	0.022	0.000	0.030	0.000
D13S317	0.000	0.401	0.018	0.000	0.002	0.159	0.014	0.000	0.030	0.000	0.049	0.000
D16S539	0.029	0.000	0.001	0.259	0.004	0.029	0.003	0.057	0.005	0.036	0.001	0.195
D2S1338	0.003	0.079	0.004	0.042	0.001	0.163	0.009	0.000	0.024	0.000	0.009	0.000
D5S818	0.035	0.000	0.001	0.288	−0.001	0.573	0.010	0.002	0.012	0.001	0.009	0.006
FGA	0.007	0.011	0.002	0.176	0.004	0.014	0.010	0.000	0.004	0.025	0.010	0.000

P value <0.003 values

**Table 7:** Fst pairwise genetic distances and corresponding P value

Central India V/S	Santal (Chotanagpur)		Oraon (Chotanagpur)		Lodha (Bengal)		Kora (Bengal)		Maheli (Bengal)		Adi Pasi (Arunachal Pradesh)	
Locus	Fst	P-value	Fst	P-value	Fst	P-value	Fst	P-value	Fst	P-value	Fst	P-value
D8S1179	0.016	0.000	0.008	0.000	0.004	0.002	0.017	0.000	0.026	0.000	0.016	0.000
D21S11	0.004	0.015	0.009	0.001	0.019	0.000	0.014	0.000	0.071	0.000	0.041	0.000
D7S820	0.020	0.000	0.028	0.000	0.017	0.000	0.001	0.208	0.001	0.278	0.021	0.000
CSF1PO	0.005	0.026	0.004	0.069	0.010	0.001	0.040	0.000	−0.003	0.960	0.018	0.002
D19S433	0.005	0.012	0.003	0.067	0.017	0.000	0.012	0.000	0.040	0.000	0.039	0.000
vWA	0.013	0.000	0.009	0.001	0.011	0.000	0.115	0.000	0.010	0.005	0.060	0.000
TPOX	0.002	0.108	0.004	0.074	0.037	0.000	0.024	0.000	0.008	0.016	0.005	0.033
D18S51	0.002	0.115	0.007	0.002	0.020	0.000	0.028	0.000	0.016	0.000	0.069	0.000
D3S1358	0.002	0.121	0.004	0.037	0.050	0.000	0.002	0.167	0.023	0.000	0.006	0.018
THO1	0.010	0.001	0.029	0.000	0.119	0.000	0.064	0.000	0.023	0.000	0.097	0.000
D13S317	0.007	0.007	0.006	0.010	0.035	0.000	0.011	0.001	0.009	0.002	0.005	0.010
D16S539	0.000	0.271	0.005	0.029	0.005	0.002	0.009	0.000	0.008	0.004	0.040	0.000
D2S1338	0.034	0.000	0.006	0.001	0.027	0.000	0.030	0.000	0.015	0.000	0.007	0.001
D5S818	0.000	0.490	0.002	0.117	0.005	0.008	0.003	0.089	0.019	0.000	0.043	0.000
FGA	0.018	0.000	0.012	0.000	0.007	0.000	0.002	0.079	0.018	0.000	0.006	0.003

P value <0.003 values

**Table 8:** Fst pairwise genetic distances and corresponding P value

Central India V/S	Bhil (Gujrat)		Sakaldwipi Brahman (Jharkhand)		Konkayastha Brahmin (Maharashtra)	
Locus	Fst	P-value	Fst	P-value	Fst	P-value
D8S1179	0.005	0.000	0.010	0.007	0.008	0.005
D21S11	0.002	0.024	0.004	0.054	0.051	0.000
D7S820	0.001	0.182	0.001	0.271	0.010	0.012
CSF1PO	0.002	0.082	−0.001	0.469	0.012	0.015
D19S433	0.001	0.142	0.007	0.019	0.003	0.073
vWA	0.005	0.001	0.002	0.155	0.006	0.040
TPOX	0.014	0.000	0.003	0.155	0.007	0.032
D18S51	0.002	0.022	0.012	0.001	0.002	0.131
D3S1358	0.000	0.309	0.005	0.072	0.004	0.129
THO1	0.001	0.131	0.032	0.000	0.000	0.395
D13S317	0.001	0.164	0.027	0.000	0.008	0.016
D16S539	0.003	0.007	0.005	0.070	0.010	0.006
D2S1338	0.005	0.000	0.010	0.004	0.010	0.003
D5S818	−0.001	0.603	0.006	0.055	−0.001	0.514
FGA	0.004	0.000	0.001	0.236	0.018	0.000

P value <0.003 Values

The central Indian population showed significant variations at maximum number of studied loci (14 out of 15) with Lodha (Bengal) and showed no significant disparity from Lambadi population (Andhra Pradesh). This finding is found consistent with the clustering pattern of observed in the NJ tree and PCA plot. We also compared central Indian population on the basis of locus wise allele frequencies at all the 15 loci with the other published populations from Palestinian_Iraq (AL-Zubaidi et al. 2014), Swedish (Montelius et al. 2008), Hungarian (Demeter et al. 2010), Shenyang_China (Hou et al. 2013), Guangdong (Zhu et al. 2005), Moroccan (Bentayebi et al. 2014), Tamil (Balamurugan et al. 2010), Croatian (Projić et al. 2007), Wallachia_ Romania (Stanciu et al. 2009), Estonia (Sadam et al. 2014) and Latvia (Jemeljanova et al. 2015) populations using Pairwise Fst distance ranging from −0.001 to 0.266 (Table 9, 10). Central Indian population showed genetic detachment from all other area specific published population data (Table 2), used for comparison and also showed significant variation at all 15 loci with Wallachia population. Central Indian population showed significant variations at 13 loci with Moroccan population and Estonian population, at 12 loci with Swedish population, at 11 loci with Shenyang population, at 10 loci with Hungarian population, at 9 loci with Latvia population and Croatian population, at 7 loci with Guangdong population and Palestinian Iraq population, at 5 loci with Tamil population (Table 9, 10). Neighbour Joining dendrogram (Fig. 2) based on Nei’s genetic distance (Nei 1972) showed genetic relatedness amongst same neighboring populations of India in the form of clustering pattern.

**Table 9:** Fst pairwise genetic distances and corresponding P value

Central Indian region V/S	Palestinian_Iraq		Swedish		Hungarian		Guangdong		Moroccan		Tamil	
	Fst	P-value	Fst	P-value	Fst	P-value	Fst	P-value	Fst	P-value	Fst	P-value
D8S1179	0.005	0.062	0.023	0.000	0.016	0.000	0.005	0.069	0.009	0.000	0.004	0.010
D21S11	0.062	0.000	0.043	0.000	0.043	0.000	0.057	0.000	0.047	0.000	0.061	0.000
D7S820	0.011	0.012	0.007	0.001	0.011	0.000	0.023	0.001	0.011	0.000	0.000	0.441
CSF1PO	0.006	0.088	0.003	0.059	0.019	0.000	0.000	0.376	0.020	0.000	0.001	0.265
D19S433	0.025	0.000	0.027	0.000	0.014	0.000	0.047	0.000	0.014	0.000	0.015	0.000
vWA	0.002	0.175	−0.001	0.849	−0.001	0.862	0.011	0.016	0.010	0.000	0.004	0.012
TPOX	0.010	0.028	0.070	0.000	0.019	0.001	0.055	0.000	0.115	0.000	0.013	0.001
D18S51	0.019	0.000	0.016	0.000	0.015	0.000	0.010	0.010	0.017	0.000	0.001	0.209
D3S1358	0.026	0.002	0.015	0.000	0.003	0.060	0.000	0.369	0.001	0.155	−0.001	0.557
THO1	0.025	0.000	0.105	0.000	0.082	0.000	0.071	0.000	0.035	0.000	0.024	0.000
D13S317	0.016	0.002	0.008	0.000	0.006	0.009	0.004	0.129	0.028	0.000	0.000	0.430
D16S539	0.005	0.095	0.011	0.000	0.010	0.000	0.010	0.022	0.014	0.000	0.002	0.090
D2S1338	0.015	0.000	0.021	0.000	0.011	0.000	0.007	0.020	0.038	0.000	0.004	0.002
D5S818	−0.002	0.623	0.007	0.004	0.006	0.009	0.091	0.000	0.009	0.000	−0.001	0.817
FGA	0.003	0.135	0.006	0.000	0.002	0.083	0.026	0.000	0.002	0.047	0.003	0.018

*NA* not available

P value <0.003

**Table 10:** Fst pairwise genetic distances and corresponding P value

Central Indian region V/S	Croatian		Wallachia		Estonian		Shenyang		Latvian	
	Fst	p-value	Fst	p-value	Fst	p-value	Fst	p-value	Fst	p-value
D8S1179	0.028	0.000	0.022	0.000	0.027	0.000	0.004	0.000	0.028	0.000
D21S11	0.067	0.000	0.050	0.000	0.041	0.000	0.058	0.000	0.047	0.000
D7S820	0.009	0.002	0.010	0.000	0.007	0.000	0.018	0.000	NA	NA
CSF1PO	0.000	0.304	0.007	0.000	0.017	0.000	0.002	0.037	NA	NA
D19S433	0.016	0.000	0.011	0.000	0.018	0.000	NA	NA	0.025	0.000
vWA	−0.001	0.671	0.002	0.001	0.002	0.017	0.085	0.000	0.001	0.066
TPOX	0.037	0.000	0.018	0.000	0.068	0.000	0.220	0.000	NA	NA
D18S51	0.004	0.031	0.011	0.000	0.015	0.000	0.152	0.000	0.141	0.000
D3S1358	0.005	0.026	0.008	0.000	0.012	0.000	0.236	0.000	0.012	0.000
THO1	0.066	0.000	0.056	0.000	0.078	0.000	0.266	0.000	0.107	0.000
D13S317	0.004	0.051	0.004	0.000	0.019	0.000	0.031	0.000	NA	NA
D16S539	0.011	0.001	0.006	0.000	0.011	0.000	0.148	0.000	0.024	0.000
D2S1338	0.021	0.000	0.017	0.000	0.028	0.000	NA	NA	0.021	0.000
D5S818	0.004	0.063	0.009	0.000	0.004	0.006	0.005	0.003	NA	NA
FGA	0.077	0.000	0.003	0.000	0.005	0.000	0.004	0.000	0.005	0.000

P value <0.003

*NA* not available

## Conclusion

The data generated here will add to the databank of various studies conducted on Indian populations. With respect to the distribution of alleles at each STR locus, all the 15 STR loci were found to be substantially polymorphic in this population. The virtue of being polymorphic makes these 15 STR loci specific and valuable in individual identification. Central Indian population showed genetic distance from all the compared (published) populations.
